# Semi-Supervised Building Extraction with Optical Flow Correction Based on Satellite Video Data in a Tsunami-Induced Disaster Scene

**DOI:** 10.3390/s24165205

**Published:** 2024-08-11

**Authors:** Huijiao Qiao, Weiqi Qian, Haifeng Hu, Xingbo Huang, Jiequn Li

**Affiliations:** 1 Department of Surveying Science and Technology, Taiyuan University of Technology, Taiyuan 030024, China; qianweiqi1190@link.tyut.edu.cn (W.Q.); huhaifeng@tyut.edu.cn (H.H.); huangxingbo3954@link.tyut.edu.cn (X.H.); lijiequn5633@link.tyut.edu.cn (J.L.); 2 Taiyuan Research Institute of China Coal Technology & Engineering Group, Taiyuan 030006, China

**Keywords:** building extraction, satellite video data, semi-supervised, disaster, optical flow

## Abstract

Data and reports indicate an increasing frequency and intensity of natural disasters worldwide. Buildings play a crucial role in disaster responses and damage assessments, aiding in planning rescue efforts and evaluating losses. Despite advances in applying deep learning to building extraction, challenges remain in handling complex natural disaster scenes and reducing reliance on labeled datasets. Recent advances in satellite video are opening a new avenue for efficient and accurate building extraction research. By thoroughly mining the characteristics of disaster video data, this work provides a new semantic segmentation model for accurate and efficient building extraction based on a limited number of training data, which consists of two parts: the prediction module and the automatic correction module. The prediction module, based on a base encoder–decoder structure, initially extracts buildings using a limited amount of training data that are obtained instantly. Then, the automatic correction module takes the output of the prediction module as input, constructs a criterion for identifying pixels with erroneous semantic information, and uses optical flow values to extract the accurate corresponding semantic information on the corrected frame. The experimental results demonstrate that the proposed method outperforms other methods in accuracy and computational complexity in complicated natural disaster scenes.

## 1. Introduction

Recently, there has been a notable escalation in the frequency and intensity of natural disasters worldwide, resulting in increased numbers of affected people and economic losses [1,2]. Buildings, as the most prominent artificial structures and geographic landmarks, serve as the primary infrastructure for human life and development [3,4,5]. Thus, accurate building extraction results are crucial for disaster monitoring, emergency responses, and damage assessments [6].

In recent years, thanks to the rapid advancement of sensor technology, the availability of monitoring data has increased from single high-resolution images to satellite video data, making building extraction available in different insights [7]. As an emerging Earth observation technology, video imaging offers the ability to capture more dynamic information than traditional single-frame imaging, featuring extensive coverage, high efficiency, and low cost [8,9]. By continuously observing a specific area to obtain a large amount of image information, it can provide new insights into surface characteristics and change processes [10]. In particular, accurately collecting information about the dynamic evolution of disaster events is crucial for formulating effective rescue plans and preventing domino effects [11]. This capability also provides valuable insights for the extraction of building information, offering different perspectives and enhancing situational awareness [12,13]. Building extraction methods can be broadly classified into two types: traditional methods and artificial intelligence (AI) methods [14,15,16,17]. 

The traditional methods for extracting buildings rely on common artificial features, making use of spectra, texture, and geometric features of buildings in remote sensing images [18]. Lin et al. [19] were the first to utilize edge detection algorithms to identify significant changes in pixel intensity at the edges of various land features and then combine them with additional information, such as roofs, walls, and shadows, to achieve effective building extraction. Turker et al. [20] proposed using the Hough transform to extract building corner points, followed by applying geometric constraints to determine the centroids and orientations of the buildings, ultimately enabling the extraction of rectangular-like structures. These methods are relatively intuitive and simple, yet they are vulnerable to complex environmental influences. Consequently, many researchers have simultaneously utilized various artificial features to extract buildings. Liu et al. [21] initially employed a region-growing algorithm for image segmentation and then extracted buildings based on spectral and geometric features. Krishnamachari et al. [22] constructed a new feature vector incorporating texture, shape, spectral, and structural features to classify each pixel for distinguishing buildings. Zhang et al. [23] proposed a novel pixel shape index designed to extract buildings by clustering homogeneous pixels that share similar shape and contour characteristics.

Although traditional building extraction methods have achieved some success, they rely on predefined features determined through laborious efforts, various rules, and prior knowledge, resulting in limited extraction efficiency and accuracy. With improvements in the resolution of remote sensing data, abundant building features become available. However, most methods based on artificial features depend solely on shallow characteristics, which are insufficient for distinguishing buildings, especially under complex natural conditions where the same ground feature may present diverse attributes [24,25]. With advancements in AI, particularly convolutional neural networks (CNNs), significant breakthroughs have been achieved in tasks such as object detection and semantic segmentation [26,27,28,29,30]. Building extraction is essentially a binary semantic segmentation task, which has emerged as a central research topic in remote sensing image processing [31]. Depending on the degree of reliance on training datasets, semantic segmentation based on deep learning can be mainly divided into strong supervised and semi-supervised approaches [32,33,34].

Strong supervised semantic segmentation methods require a substantial number of annotated training samples to learn features [35]. These models are pre-trained using this knowledge to ultimately extract buildings [36]. Alshehhi et al. [28] were among the first to introduce CNNs into the field of automatic building extraction, employing a sliding window strategy to achieve an outstanding performance in extracting buildings from aerial imagery. Fully convolutional neural networks (FCN) are emerging networks that can achieve end-to-end prediction in a new and efficient way [37]. Built upon FCN, many networks for building extraction have emerged and been refined from various perspectives. Zheng et al. [38] utilized UNet with a skip connection as a backbone and embedded cross-layer blocks to optimize the precision of building extraction. Chen et al. [29] utilized DeeplabV3 as the backbone and combined it with residual networks (ResNet) to further optimize the extraction results of buildings. Although strong supervised methods achieve better building extraction performance, these neural network models require a large number of training samples and are sensitive to errors in the training data, which limit their wide utilization in natural disaster scenes.

To simplify the labeling process of training samples and enhance the accuracy of building extraction, researchers have proposed semi-supervised learning strategies. These methods combine limited manually labeled data with weakly labeled data generated by unsupervised classification methods for model training, thereby reducing the cost of acquiring accurately labeled data [39,40,41]. Chen et al. [42] proposed a semi-supervised semantic segmentation model based on cross-pseudo supervision, in which both labeled and unlabeled images are fed into two segmentation networks with the same structures but different initializations. Hong et al. [43] efficiently expanded a few labels into considerable labeled samples for image classification by using a cross-modal deep learning algorithm. Another famous method learns deep features from labeled data by means of specific loss functions [44], generative adversarial networks (GANs) [45], and other contrastive learning methods [46] to generate pseudo-labels of unlabeled data. Wu et al. [47] generated pseudo-labeled remote sensing samples to enhance the segmentation performance of deep learning models on small labeled data.

These methods significantly reduce the need for a large number of accurately labeled samples, and they maximize the use of valid samples to improve the accuracy of building extraction [48]. However, there are still several shortcomings in the field of semi-supervised building classification that need to be explored.

(1) Training data comprise manually labeled building data, which are susceptible to irregular distribution and diverse sensor outputs, and weak samples, whose generation models are complicated. The challenges lead to delays in acquiring training data, thereby hindering the extraction of building information critical for natural disaster emergency responses.
(2) These methods rely on a limited number of manually labeled samples and newly generated weak samples for learning, lacking explicit mechanisms to effectively extract valid data from the weak samples generated anew and giving less consideration to the impact of the accuracy and quality improvement on the weak samples on the subsequent model classification.

To better address problems of the weak generalization ability of the model and difficult, quickly obtained training datasets, this paper proposes a new method that is composed of a prediction module and an automatic correction module. The prediction module outputs the initial building extraction results of input images based on a typical semantic segmentation network, and then the automatic correction module takes the output of the prediction module as input. By correcting the semantic segmentation information based on displacements between the frame with erroneous pixels and the adjacent corrected frame, the accuracy of building extraction is ultimately further improved.

The contributions of this paper mainly include the following three aspects.

(1) This paper proposes a new method composed of the prediction module and the automatic correction module for accurate and complete building extraction in satellite video data. The experimental results on the Indonesia tsunami video data show that our method outperforms the basic semantic segmentation algorithm in terms of the speed of building extraction and the accuracy of complex building footprints.
(2) The displacements are firstly designed as key units to optimize the initial building extraction results in the automatic correction module. This approach relies solely on pixel movement between adjacent video frames without additional data or algorithms, enabling high-precision building extraction from satellite video data in disaster scenarios.
(3) The novel automatic correction module significantly improves the performance of the base network by harnessing the characteristics of video data in natural disaster scenarios and the relationships between adjacent frames. It eliminates the need for a large amount of manually labeled data or complex models to generate weak samples. This method is easy to operate and can be readily applied to other deep learning models or scenarios that require timeliness and complexity.

The following sections present the proposed method and the experiments performed. Section 2 elaborates on the proposed method. Section 3 provides the experiments and analysis, which explains in detail our comparative experiments and analysis of the experimental results. Section 4 discusses our method. Finally, Section 5 concludes the paper.

## 2. Materials and Methods

**Figure 1:**
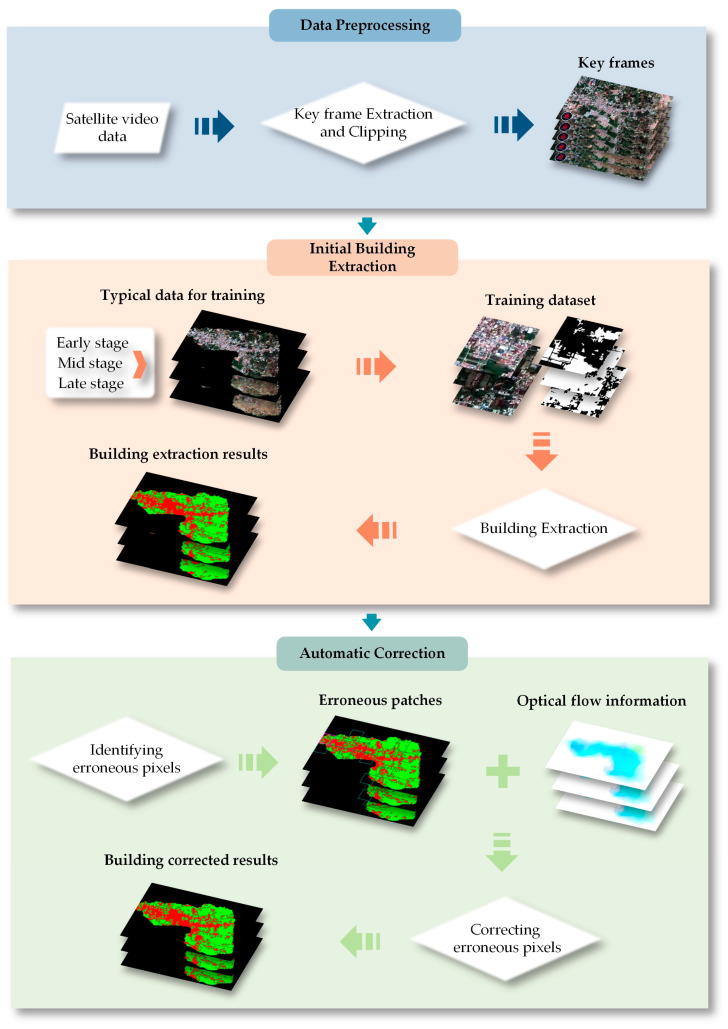
The framework of the semi-supervised building extraction.

In this work, we propose a semi-supervised method to extract buildings from natural disaster scenarios using satellite video data based on deep learning and automatic correction. An overview of the proposed semi-supervised building extraction framework is illustrated in Figure 1.

Firstly, the satellite video images are preprocessed, and key frame extraction is used to speed up the calculation. Then, an off-the-shelf semantic segmentation network based on deep learning is used to extract the preliminary buildings, where initial training labels are acquired through a supervised classification method and data augmentation algorithms based on extremely small amounts of video data and treated as inputs to train the network. The outputs of the deep neural network are the semantic segmentation maps that correspond to the building labeling results of the satellite video’s key frames. Finally, the most important step is automatic correction, which identifies erroneous pixels in the semantic segmentation results obtained from the previous step. To enhance the accuracy of building extraction in the semantic segmentation results obtained from the previous step, an automatic correction approach for erroneous pixels was adopted as the final step. This approach involves two main steps: automatically identifying the wrong pixels and correcting them by using motion information derived from adjacent video key frames.

### 2.1. Data Preprocessing

Initially, the experimental data contained a video rather than single-frame images, making it challenging to input them into the building extraction model directly. In order for the experimental data to adapt better to the proposed algorithm, there are a series of preprocessing operations. Thus, data preprocessing includes converting the video into multi-frame images, filtering out frames with undesirable artifacts, and key frame extraction and cropping.

### 2.2. Initial Building Extraction Based on SegNet

**Figure 2:**
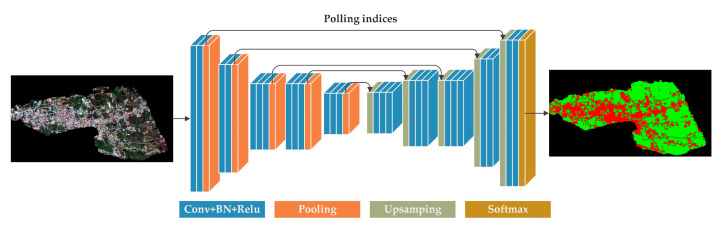
The architecture of SegNet.

The advent of convolutional neural networks has revolutionized semantic segmentation, enabling the automatic generation of geo-localized semantic class maps for each pixel from the earth observation data. This includes the classification of diverse elements such as buildings, vegetation, and more [43,44,45]. SegNet, published in 2017 TPAMI, notably boasts more than 1800 citations and achieves the highest average accuracy, class average accuracy, mean intersection over union (mIoU), and boundary F1 measures on two popular datasets: the CamVid dataset and the SUN RGB-D dataset, surpassing FCN, DeepLabv1, and DeconvNet [49]. As shown in Figure 2, the encoder consists of 13 convolutional layers from VGG-16, followed by max pooling operations. In the decoder, upsampling and convolutional operations were performed corresponding to the encoder layers. Finally, a multi-class softmax classifier was utilized to predict the class at the pixel-wise level.

SegNet is a supervised network and requires a large number of training data with pixel-level annotations used as prior knowledge to obtain high accuracy. To efficiently acquire the building extraction results of SegNet in disaster scenarios, utilizing the limited data available in the current scene to create the training dataset is a viable choice.

In the context of disasters, there are three distinct phases: early, mid, and end. The early stage follows the occurrence of the disaster, characterized by minor changes in the displacement rather than appearance of objects nearest to the disaster-affected area. The mid phase refers to the period when significant changes in both displacement and appearance have been observed. In the final phase, the power of the tsunami has greatly weakened, leaving ground objects mostly in a state of disrepair and destruction, with only minor changes at the level of displacement. Therefore, three key frames representing three distinct phases will be selected as the original training samples, and their corresponding labels will be achieved through supervised classification based on a support vector machine (SVM) in a binary manner. This approach enables the generation of the original training dataset, but it cannot meet SegNet’s training requirements to achieve high-accuracy results. Thus, some common algorithms for data augmentation are employed to expand the training dataset. 

### 2.3. Automatic Correction

It has been widely recognized that achieving satisfactory results by inputting a small number of training datasets into any supervised network is challenging, particularly for building extraction [50,51,52,53]. Because the existing building datasets are not only limited in quantity but also their diverse styles and distinct materials, this leads to heterogeneity of the building data. Moreover, natural disaster scenes are highly complex, further complicating the task of building extraction. Thus, a novel automatic correction algorithm is proposed as a supplement to the initial building extraction results obtained from SegNet. This algorithm entails the automatic identification of the semantic segmentation results corresponding to the erroneous pixels and erroneous frames, followed by an automatic correction process.

#### 2.3.1. Identification of Erroneous Pixels

In a natural disaster scene, the types and variations in inaccuracies are diverse and complex, thus, it needs a step-by-step approach to address them effectively. To improve precision and optimize the process of identifying different types of variations in building areas, sliding window technology is employed, through which each area with evaluation exclusively captures a single type of building area variation. Specifically, the size of the sliding window is set to 190×170 pixels, determined through a comprehensive consideration of the building area and computational complexity after numerous trials, resulting in 60 patches.

Firstly, we determined the type of variation in the building area.

Through a comprehensive comparative analysis of the initial building extraction results, it can be seen that there are two principal types of alterations in the area of buildings across adjacent key frames. The first type, a decreasing type that is easily understood, presents a reduction in building footprints, where buildings are obliterated by the tsunami. The second type, an increasing type, conversely signifies an expansion characterized by the inherent structures alongside the displacement and accumulation of rubble from destroyed or damaged buildings. The area difference in the selected patch between the first key frame and the last is calculated as:

(1) $$diff=S_{first}-S_{last}$$

(2) $$S=\left\{\begin{array}{ll} S+1, & \mathrm{if}\;p[i,j]=(255,\;0,\;0)\\ S, & \mathrm{otherwise} \end{array}\right.$$

where $S_{first}$ and $S_{last}$ are the building’s area of the first frame (frame 179 in this research) and last frame (frame 449 in this research) for the selected patch, diff represents their areas’ difference. If the area difference is less than 0, it indicates that the area of buildings in the first frame surpasses that in the last one, thereby classifying it as the first type. Conversely, it should be greater than 0, and this is marked as the second type.

S denotes the area of buildings (the number of building pixels) within a chosen patch, with i and j representing the row and column indices corresponding to the patches. The maximum dimensions of i and j are 190 and 170, respectively. The parameter p is employed to judge the area occupied by the buildings within the selected patch. In our study, red represents buildings, which is denoted by RGB (255,0,0). Therefore, pixels exhibiting a value of 255 in the first channel are tallied, and the cumulative result is considered as the pixel count for buildings, effectively measuring the buildings’ area within the designated patch.

Next, we identified the erroneous pixels and corresponding key frames.

**Figure 3:**
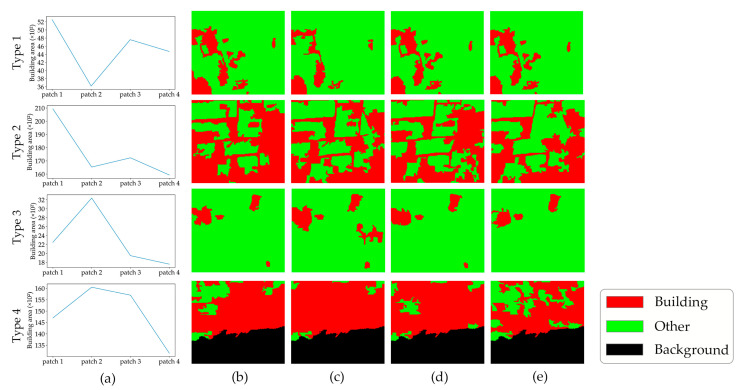
Summary of error types in the area-decreasing category: (**a**) change curve of the building area; (**b**–**e**) initial building extraction results of four adjacent frames.

**Figure 4:**
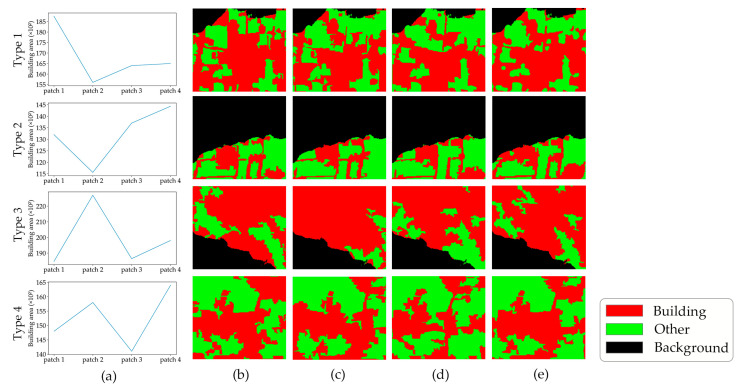
Summary of error types in the area-increasing category: (**a**) change curve of the building area; (**b**–**e**) initial building extraction results of four adjacent frames.

Following the general criteria, erroneous pixels, along with the key frames and their corresponding semantic segmentation results to which they are attached, need to be further extracted. Thus, four patches from the same row and column of four sequential frames (*S_fm_*, *S_fm+1_*, *S_fm+2_*, and *S_fm+3_*) are grouped together and inputted to determine the changes in the building area. If the change follows the monotonically increasing or decreasing trend, indicating no errors in this group, then it proceeds to the adjacent patch for the next iteration. If there is turbulence in the trend, it indicates potential errors within this group. Summarizing these turbulent trends, each type of variation identified in the results of the first step includes four types of errors, as illustrated in Figure 3 and Figure 4.

**Figure 5:**
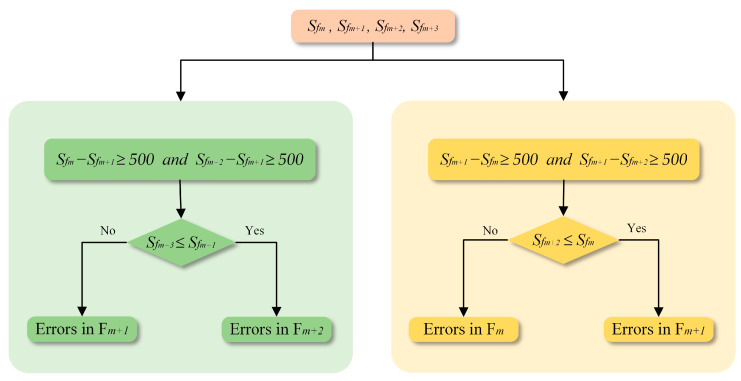
Criteria for evaluating types of decrease.

As Figure 3 shows, when the selected patch belongs to the decreasing type, the rules for identification of the incorrect frame with an erroneous part can be divided into two categories, and the criteria judgment is shown in Figure 5.

(1) As illustrated by type 1 and type 2 in Figure 3, a declining trend, succeeded by an ascending trajectory across the first three frames, necessitates the inclusion of the fourth frame for detailed examination. The criterion for conditional judgment is specified on the left side of Figure 5, and the decision is as follows:

(3) $$diff>0\;and\;S_{fm}-S_{fm+1}\geq 500\;and\;S_{fm+2}-S_{fm+1}\geq 500$$

After extensive experimentation, we found that a threshold value of 500 produced the best results. If this value is too large, it can lead to missed detection of erroneous regions, whereas if it is too small, it can cause false detection of erroneous regions. This enhances the identification of pixels most likely affected by incorrect semantic results.

The variation in building area between the fourth and second frames acts as a critical determinant for pinpointing errors within the image sequences. As Formula (4) describes, a positive difference is indicative of errors in the semantic result of the second frame. In contrast, a value less than zero highlights the deficiencies of building extraction in the third frame.

(4) $${Frame}_{error}=\left\{\begin{array}{ll} {Frame}_{m+2}, & if\;S_{fm+3}\leq S_{fm+1}\\ {Frame}_{m+1}, & \mathrm{otherwise} \end{array}\right.$$

(2) Depicted by type 3 and type 4 in Figure 3, the area of the building in the selected patch presents an increasing and then decreasing trend across the first three frames in this group. Therefore, the third frame requires a more detailed analysis, as shown on the right side of Figure 5. The judgment formula is as follows:

(5) $$diff>0\;and\;S_{fm+1}-S_{fm}\geq 500\;and\;S_{fm+1}-S_{fm+2}\geq 500$$

Comparing the area of the building between the third and first frames, if the difference is less than or equal to zero, the errors are attributed to the pixels in the second frame; otherwise, should the difference be greater than zero, the incorrect pixels are identified within the first frame. Specifically, the comparing method is formulated, as Equation (6) shows.

(6) $${Frame}_{error}=\left\{\begin{array}{ll} {Frame}_{m+1}, & if\;S_{fm+2}\leq S_{fm}\\ {Frame}_{m}, & \mathrm{otherwise} \end{array}\right.$$

**Figure 6:**
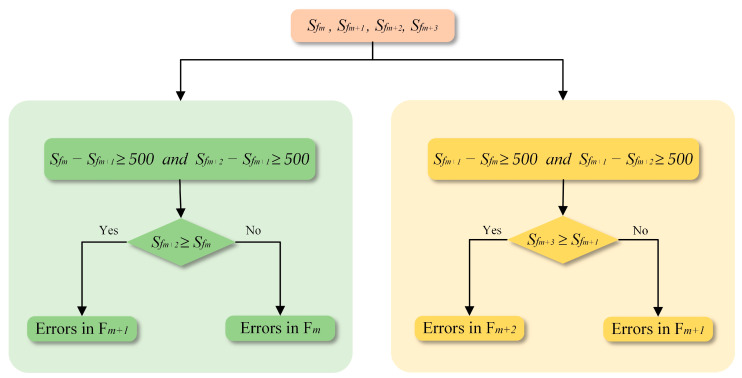
Criteria for evaluating types of increase.

As Figure 4 demonstrates, when the area of buildings in the selected patch presents an increasing trend, the rules for determining the incorrect frame can also be divided into two categories, and the details are shown in Figure 6.

(1) If the building area in the selected patch demonstrates a decreasing and then increasing trend across the first three frames in this group, as depicted by type 1 and type 2 in Figure 4, the third frame’s image necessitates a more granular analysis. The criterion for conditional judgment is on the left side of Figure 6 and is specified as follows:

(7) $$diff<0\;and\;S_{fm}-S_{fm+1}\geq 500\;and\;S_{fm+2}-S_{fm+1}\geq 500$$

Comparing the area of the building between the third and first frames, if the difference is greater than or equal to zero, the errors are attributed to pixels in the second frame. Otherwise, if the difference is smaller than zero, the incorrect pixels are identified within the first frame. More precisely, the comparing method is formulated as:

(8) $${Frame}_{error}=\left\{\begin{array}{ll} {Frame}_{m+1}, & if\;S_{fm+2}\geq S_{fm}\\ {Frame}_{m}, & \mathrm{otherwise} \end{array}\right.$$

(2) If an initial increasing trend is followed by a decreasing one, as demonstrated by type 3 and type 4 in Figure 4, observed across the first three frames, the fourth frame needs to be included for further evaluation. The criterion for conditional judgment is on the right side of Figure 6, and the detailed criteria are expressed as:

(9) $$diff<0\;and\;S_{fm+1}-S_{fm}\geq 500\;and\;S_{fm+1}-S_{fm+2}\geq 500$$

The difference in building areas between the fourth and the second frames serves as a decisive factor for identifying errors. As outlined in the right side of Figure 6, a difference greater than or equal to zero signifies the errors of semantic results in the third frame. Conversely, a negative difference indicates a flaw in the building extraction result for the second frame (detailed as shown in Equation (10)).

(10) $${Frame}_{error}=\left\{\begin{array}{ll} {Frame}_{m+2}, & if\;S_{fm+3}\geq S_{fm+1}\\ {Frame}_{m+1}, & \mathrm{otherwise} \end{array}\right.$$

After identifying the aforementioned erroneous regions p, we marked them simultaneously in both the semantic segmentation result $S_{e}$ and its corresponding image frame $F_{e}$.

#### 2.3.2. Automatic Correction Process

For the above erroneous pixels, a corresponding correct algorithm was successively implemented. The adjacent frames in the experimental video, along with the corresponding semantic segmentation results, are similar, and the minor differences between them are motion changes, other than obvious appearance changes. Thus, the adjacent frames can be converted to each other once the motion information is achieved. 

Optical flow is the instantaneous velocity of the pixel movement of a spatially moving object on the observation plane [54]. When the time interval is extremely brief, the optical flow corresponds to the displacement of pixels. Optical flow estimation methods are designed to find the corresponding relationship between the previous frame and the current frame by using the change in pixels in the time domain and the correlation between adjacent frames [55,56] so as to calculate the motion information of objects between adjacent frames. 

**Figure 7:**
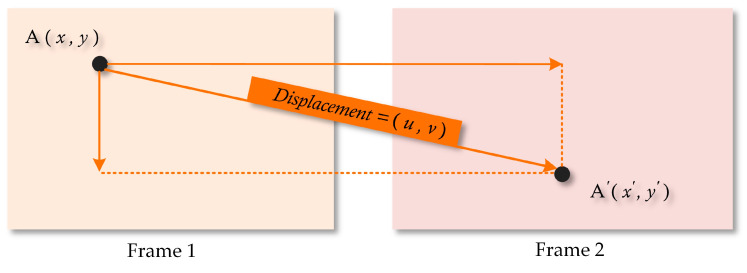
Illustration of the displacement computation.

As shown in Figure 7, A is a sample pixel located in frame 1, while A’ represents the corresponding pixel of A in frame 2, attributed to some motion. The displacement vector characterizes A’s movement in both the x and y directions, quantifying both magnitude and direction. The coordinate information between A and A’ is relevant, facilitating their interconversion based on the displacement. The interconversion relationship is formulated as follows:

(11) $$\left\{\begin{array}{c} x^{\prime }=x+u\\ y^{\prime }=y+v \end{array}\right.$$

In the automatic correction process, there are numerous computational elements with redundant and confusing names. To clearly describe the automatic correction process, a list of abbreviations and their corresponding definitions is provided in Table 1. The coordinates of the erroneous pixels in P are recorded as ($x_{i}$, $y_{j}$), with ranges of  i and j being [0, 190] and [0, 170], respectively. Then, the motion information of each erroneous pixel between $F_{e}$ and $F_{c}$ is computed using the optical flow-based adaptive thresholding segmentation (OFATS) algorithm [57], denoted as ($u_{i}$, $v_{j}$). In this context, OFATS employs the optical flow estimation module, FlowNet 2.0, which has been validated with an RMSE of 0.30 pixels horizontally and 0.11 pixels vertically [58]. This accuracy ensures the provision of reliable motion information. 

**Figure 8:**
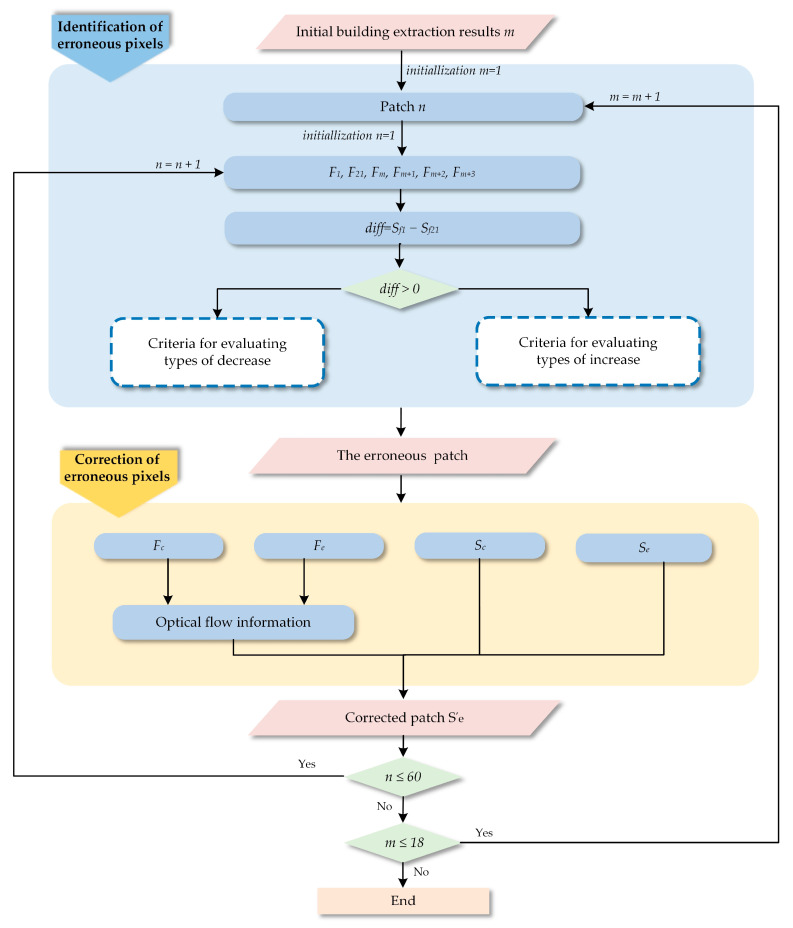
The flowchart of the proposed automatic correction algorithm.

As depicted in Table 1, the relevant coordinate information of the erroneous region P in $F_{c}$, represented as (${x^{\prime }}_{i}$, ${y^{\prime }}_{j}$), can be derived from the coordinates ($x_{i}$, $y_{j}$) and optical flow values ($u_{i}$, $v_{j}$). Subsequently, the semantic information corresponding to coordinates (${x^{\prime }}_{i}$, ${y^{\prime }}_{j}$) is extracted from $S_{c}$. This information is then assigned to the corresponding pixels in the erroneous regions P of $S_{e}$, thus completing the correction process for $S_{e}$. When the automatic correction of this patch is completed, the adjacent patch within the four adjacent frames undergoes another round of the specified criteria. Once 60 small patches are completed in an iterative manner, another set of four adjacent frames is selected to execute a new round of the same criteria. Specifically, the second frame in the current iteration is reused as the starting frame, and the subsequent three adjacent frames are grouped in turn for a new round of determination. As shown in Figure 8, following this process allows for the completion of automatic correction for the initial semantic segmentation results of all key frames.

## 3. Results

### 3.1. Dataset and Computing Environment

The experimental data are a synthetic video shoot by Digital Globe’s WorldView (Longmont, CO, USA), which captures the total natural disaster change progress in Petobo, Indonesia, where a 7.5 magnitude earthquake trigged a tsunami on 28 September 2018. After converting this video into sequence frames, there are 301 sequence frames when removing the invalid data, such as titbits data, at the beginning of the video as well as artificially added buffer data. 

**Figure 9:**
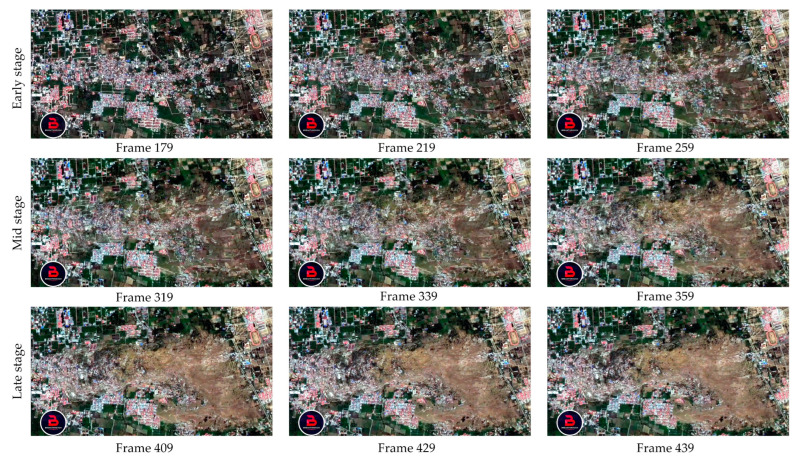
Representative schematic of the preprocessed key frames.

Notably, there is a redundancy between the adjacent video images (changes in the pixels or objects only appear in disaster-stricken areas), but the pixels that remain static amount to approximately half of each frame, which hampers efficient building extraction. Thus, a clustering algorithm was employed to identify the key frames, resulting in the extraction of 21 key frames; the OFATS method was utilized to divide the changed areas from unchanged parts of these key frames. The representative preprocessing results are shown in Figure 9. For this tsunami event, three distinct phases can be identified: the early phase, mid phase, and late phase. During the early stage, there are no significant changes in shape, but there is a slight motion observed for the buildings, as depicted in frames 179 to 259. For the mid phase, the buildings underwent significant changes in both appearance and motion, with many buildings being destroyed, as evidenced in frames 319 to 359. For the late phase, objects exhibited continued motion changes, characterized by the accumulation of debris and rubble. As the tsunami’s impact weakened, it was difficult to observe significant changes in the shape, as evidenced from frames 409 to 439.

In this study, the experimental setup utilized a server equipped with 128 GB of memory, an Intel Xeon(R) E5-240 CPU, and a TITAN V GPU. The server ran on the Linux operating system, specifically Ubuntu 20.04 LTS, and primarily utilized the Pytorch framework version 2.3.0, as outlined in Table 2.

### 3.2. Results

#### 3.2.1. Construction of the Training Dataset

SegNet is a supervised semantic segmentation network whose performance heavily relies on the availability of extensive labeled datasets. However, the rapid extraction of buildings is the most important thing in disaster relief; therefore, three frames that could characterize different periods of the disaster were selected as the basic data, and then a series of data augmentation operations were conducted to obtain enough training data with a small computational cost.

**Figure 10:**
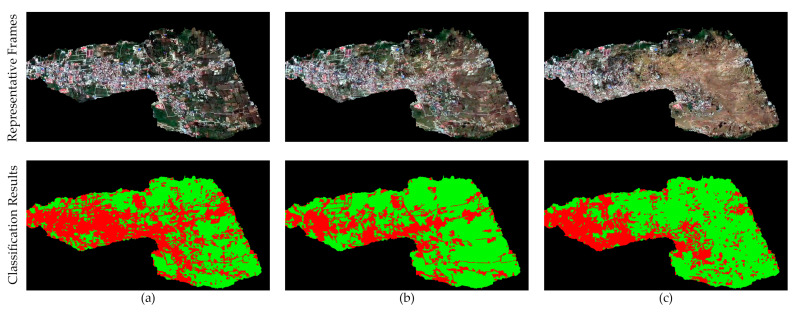
Representative images and supervised classification results across various phases of the disaster: (**a**) early phase; (**b**) mid phase; (**c**) late phase. The red and green channels in the supervised classification results denote buildings and others, respectively.

The selected three frames are frame 219, frame 319, and frame 409, corresponding to the different disaster phases, while the supervised classification results based on SVM are the ground truth. The classification results visually align with the developmental and transitional patterns of the natural disaster, demonstrating a trend where buildings become increasingly fragmented from the early to late phases (as Figure 10 shown).

As summarized in Table 3, the performance of supervised classification across different time frames reveals that the overall accuracy (OA) for all datasets exceeds 95%, while the user’s accuracy (UA) for building identification consistently surpasses 98% across all frames. Frame 219 achieves an OA of 97.54% with a kappa coefficient of 0.95. The building class exhibits a UA of 98.24% and a producer’s accuracy (PA) of 97.51%. In comparison, the other land features class demonstrates a UA of 96.62% and a PA of 97.61%. In frame 319, the classification accuracy is higher, achieving an OA of 99.26% and a kappa coefficient of 0.98. The UA and PA for the building class are 99.71% and 99.16%, respectively, and for the other land features class, the UA and PA are 98.42% and 99.45%, respectively. However, in frame 409, although the OA slightly decreases to 96.60% with a kappa coefficient of 0.93, the UA for the building class remains high at 99.93%. These findings indicate that the supervised classification results are consistently accurate across different time frames, demonstrating high reliability for their use as ground truth in subsequent semantic segmentation tasks.

**Figure 11:**
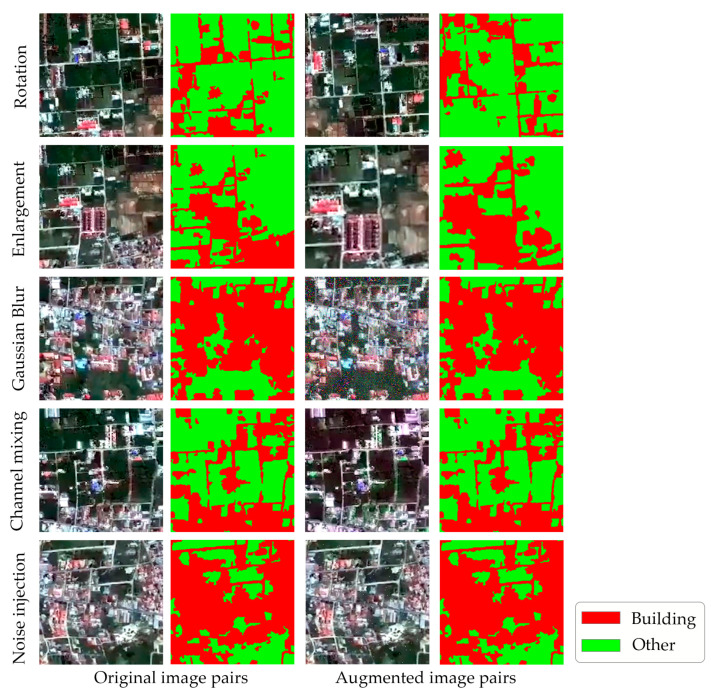
Example images of data enhancement in the training dataset.

In order to meet the needs of the experimental environment and make the research run smoothly, these frames and corresponding ground truths are split into small images of 256 × 256 size. Subsequently, extensive data augmentation techniques are applied, and some representative samples are shown in Figure 11.

Extensive data augmentation techniques include rotation, scaling, flips, blur, gaussian blur, gamma correction, RGB channel mixing, noise injection, and translation. These operations facilitate the generation of 20,000 pairs within the dataset. The above 20,000 data pairs are divided into training and test datasets in a ratio of 7:3, which ensures coverage of data from various periods of the disaster, thereby preventing any artificial imbalances that could affect the accuracy of the SegNet model.

#### 3.2.2. Initial Building Extraction Results

The training dataset is inputted into SegNet for pre-training, while the test dataset is utilized to evaluate the outcomes derived from the pre-trained SegNet model. From the test images, three frames with initial building extraction results standing for different disaster phases are randomly selected to ensure accuracy verification.

**Figure 12:**
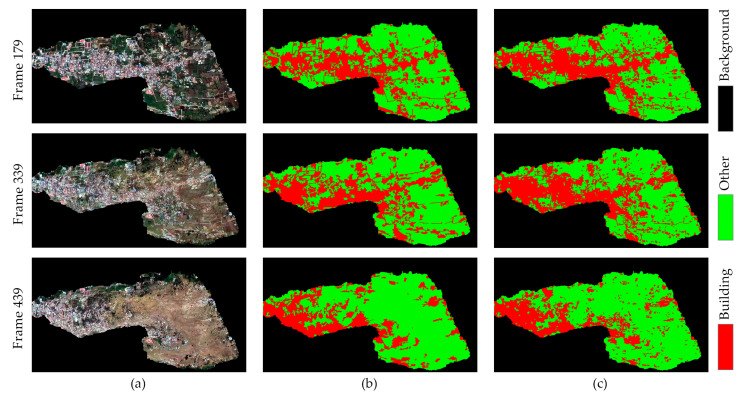
Initial building extraction results for the three frames: (**a**) original image; (**b**) initial building extraction results; (**c**) ground truth.

As shown in Figure 12, frames shot in different periods obtain varied building extraction results in shape and size, which differ from the corresponding ground truth. The pre-trained SegNet is prior to extracting linear objects, but the model has weak robustness regarding sporadic and broken buildings, especially when the impact of the tsunami is more severe.

For the accurate evaluation of the building extraction results using the pre-trained model, Table 4 shows the accuracy metrics across three frames in different disaster periods. The model performs best on frame 179, achieving a kappa coefficient of 0.73 and an OA of 87.48%. Specifically, the UA for buildings in frame 179 is 88.51%. In frame 339, the OA decreases to 83.67% with a kappa coefficient of 0.65, and the UA for the building class drops to 82.93%. Frame 439 shows an OA of 85.67% and a kappa coefficient of 0.63. However, the UA for the building class further decreases to 74.10%, while the UA for the other class increases to 90.02%. Notably, the PA for building recognition in frames 339 and 439 is 75% or lower, indicating a higher likelihood of misclassification for damaged and isolated buildings as the disaster progresses. These results highlight the increasing difficulty in accurately extracting buildings in the later stages of the disaster, underlining the need for improving the methods that handle the complex conditions encountered in these periods.

#### 3.2.3. Automatic Correction of Building Extraction Results

The initial building extraction results illustrate that the buildings on different phases can be initially extracted using a pre-trained SegNet based on limited training data. Still, it cannot be ignored that the PA and extraction accuracy of buildings are smaller than 80%, and some isolated and fragmented buildings are difficult to identify from the mid to late stages of the disaster. If these types of objects cannot be accurate identification, it is hard to effectively prevent secondary disasters and strictly determine the disaster’s magnitude. Thus, an automatic correction algorithm was applied to improve the accuracy of building extraction based on these primary semantic segmentation results.

**Figure 13:**
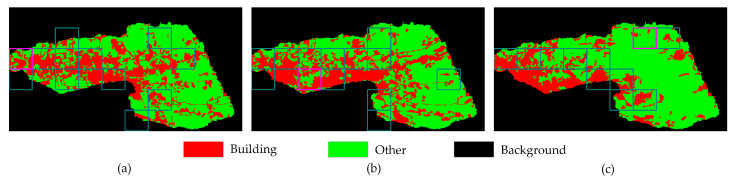
Identification results of erroneous parts in the semantic segmentation results of diverse frames: (**a**) early phase: frame 179; (**b**) mid phase: frame 339; (**c**) late phase: frame 439.

Figure 13 shows the erroneous patches of three initial semantic segmentation results. To illustrate the process of identifying and correcting these errors in detail, three patches with inaccuracies are highlighted. These representative erroneous areas are selected based on the evolving characteristics of ground objects during the disaster period. 

Frame 179, belonging to the early stage, exhibits minimal impact from the tsunami, with only slight displacements observed. Among the twelve highlighted erroneous parts in blue, one particular area, highlighted in rose (Figure 13a), serves as an example of correction despite being far from the disaster-affected regions. In the semantic segmentation result of frame 339, there are 13 incorrect patches. The rose-colored area in Figure 13b is seriously affected by the disaster, exhibiting a diverse range of textures and spectral characteristics in the buildings, which align with the typical changes observed during the mid phase of the disaster. Consequently, this sub-region was chosen as a representative sample to showcase the automated correction algorithm. For the late phase, the semantic segmentation results of frame 339 reveal 11 erroneous parts, as depicted in Figure 13c. The rose-colored area, located in the top-right corner, exhibits fragmented buildings, which is characteristic of this stage.

To systematically explain the automatic correction process for the three samples, the details are presented in Table 5. Identifying erroneous information involves two steps: Firstly, determine the change trend based on the area difference between the first frame and the last frame of the selected patch. The differences between frame 179 and frame 339 show an increasing trend, as they are both smaller than zero. However, for frame 439, the difference is greater than zero, suggesting a decreasing trend. Next, locate the image information containing the erroneous pixels by comparing the area differences of adjacent key frames.

Erroneous pixels are present in frame 179, marked as $f_{1}$, which is the first frame among all key frames. The corresponding semantic segmentation information is marked as $S_{f1}$. Frame 199, labeled as $f_{2}$, is used as the adjacent frame used for correction. Similarly, frame 339—designed as $f_{10}$—contains erroneous pixels in the tenth frame among all key frames. Its corresponding semantic segmentation information is identified as $S_{f10}$, utilizing frame 329 (labeled as $f_{9}$) as the adjacent frame for correction. Furthermore, the presence of frame 439, labeled as $f_{19}$, exhibits erroneous pixels in the nineteenth frame among all key frames. Its corresponding semantic segmentation information is represented as $S_{f19}$ with frame 429 (labeled as $f_{18}$) serving as the adjacent frame for correction.

Error correction primarily involves rectifying the erroneous pixels based on the corrected images: Firstly, extract the coordinates N of the erroneous pixels and then calculate the optical flow values I between the corrected frame and the frame with erroneous pixels using the OFATS algorithm to obtain the coordinates $N^{\prime }$ on the corrected images. Next, the coordinates are used to extract the semantic segmentation information from the corresponding semantic segmentation for correction $S_{c}$ based on $N^{\prime }$; then, replace the semantic segmentation information of the erroneous pixels.

**Figure 14:**
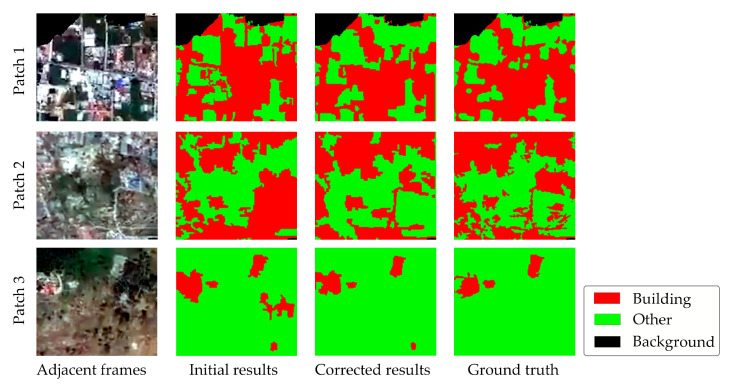
Correction process for highlighted area in rose from Figure 13.

The results before and after applying the correction algorithm for the three example areas are shown in Figure 14. In these images, over-classified pixels in error-prone areas are effectively removed, and under-classified pixels are supplemented. Consequently, the corrected images align more closely with the ground truth, and the accuracy of the pixels has visibly improved.

**Figure 15:**
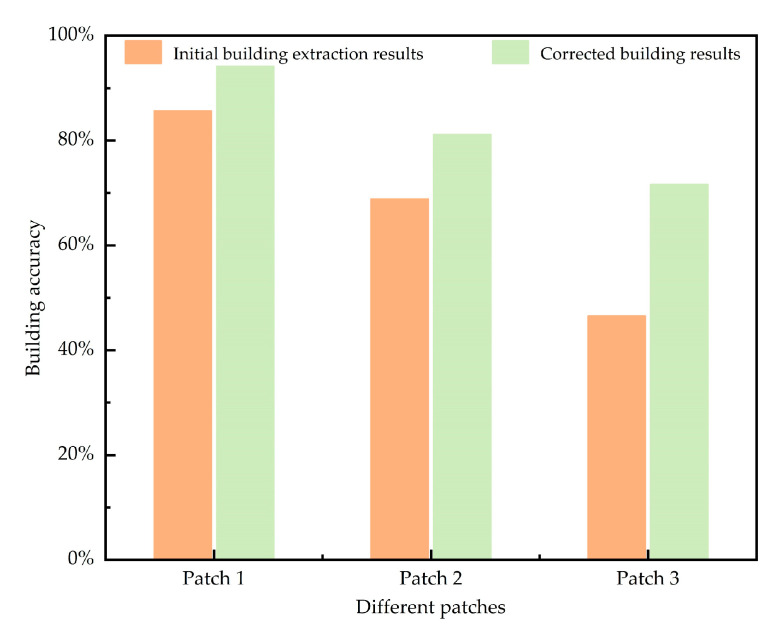
Comparison of accuracy before and after calibration of example area.

For the highlighted area, Figure 15 further illustrates the comparison of accuracy before and after correction. 

After applying the correction module to the example areas, there is a significant improvement in building accuracy across all regions, with an average increase of 15%. Specifically, the accuracy in patch 1 increases from 85.6% to 94.2% (an improvement of 8.6%), and in patch 2, from 68.8% to 81.1% (an improvement of 12.3%). Patch 3, which is imaged during the post-disaster phase, sees an accuracy increase of 25.1%, from a lower initial accuracy due to a substantial tsunami impact that significantly altered the basic properties of buildings to an improved accuracy of 71.6% after correction. Based on the samples’ results, it is evident that the accuracy of building extraction has significantly improved following the application of the correction algorithm.

The accuracy of the automatic correction algorithm is verified not only for the small highlighted area but also for the whole image. Across the frames, the automatic correction model’s performance achieves a satisfying OA (as shown in Table 6). In frame 179, the OA is 89.28%, with the building class achieving a UA of 87.82% and a PA of 85.15%. For frame 339, the model exhibits an OA of 86.62%, whereas the building class records a UA of 82.40% and a PA of 84.39%. In frame 439, the OA reaches 87.99%, while the UA and PA for the building class are 82.09% and 76.12%, respectively. These results indicate the model’s robustness across different disaster periods despite some variations in the building class accuracies.

**Figure 16:**
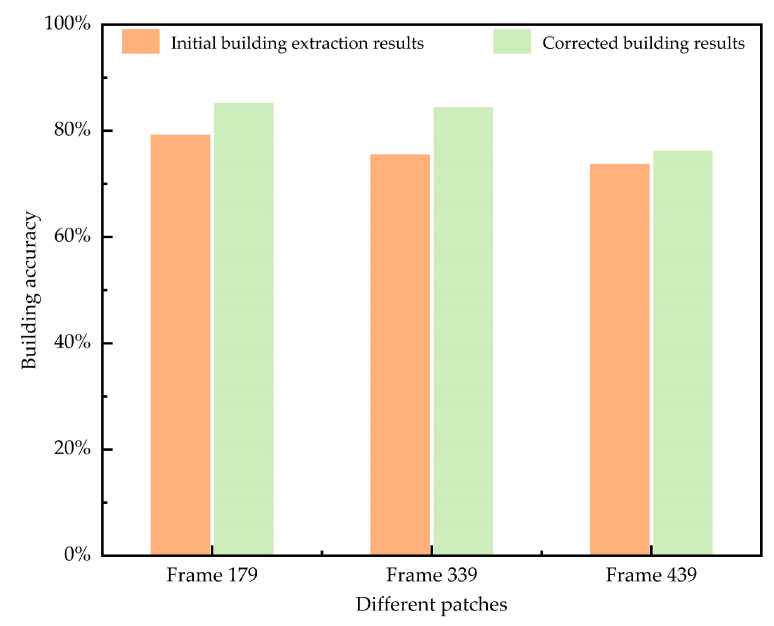
Comparison of accuracy before and after calibration of example frames.

A deeper analysis of the experimental results can be facilitated by comparing the quantitative performance metrics from different operations. Figure 16 illustrates the building extraction accuracy of the three-frame images before and after correction, showing an increase in accuracy across all frames to varying degrees. Specifically, the building extraction accuracy of frame 179 improves from 79.15% to 85.15%, an increase of 6%. This improvement is related to the early stage of the disaster, where changes in the buildings are not very noticeable, making extraction relatively easier. For early disaster data, this algorithm functions as a correction for random errors to some extent, which is equivalent to correcting these initial results with multiple highly similar corrected data. For frame 339, the accuracy increases significantly from 75.45% to 84.39%, representing an 8.94% improvement. This substantial increase is attributed to the mid-disaster period, during which buildings are undergoing drastic changes, leading to a more homogeneous spectrum and a higher likelihood of misclassification. Thus, the accuracy improvement is more pronounced as the correction algorithm uses adjacent accurate building extraction results to rectify the erroneous data. The accuracy of frame 439 increases by 2.49%, from 73.63% to 76.12%. In the late disaster stage, the number of intact buildings has significantly increased, and the morphology of the remaining structures is ambiguous. Extracting complete buildings from a single frame is challenging. While using adjacent frames offers some improvement in accuracy, the benefit is limited due to the information loss in these neighboring frames.

Overall, after applying the correction algorithm, there has been a notable enhancement in the precision of building extraction, effectively meeting the requirements for extracting emergency disaster information.

#### 3.2.4. Comparison of Building Extraction Results

**Figure 17:**
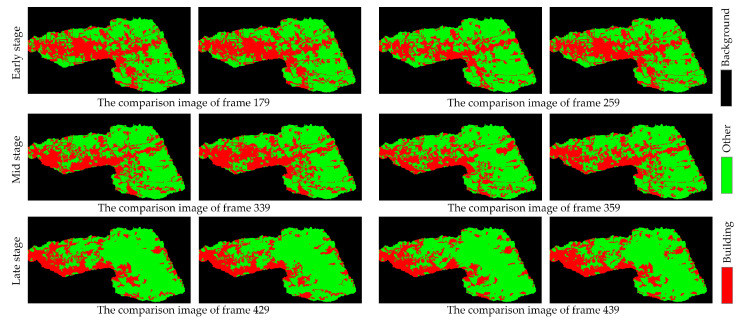
Visualization of initial and corrected semantic segmentation results on representative key frames.

All key frames have been corrected using the automatic correction algorithm. The representative initial and corrected building extraction results are shown in Figure 17. 

Most of the initial results have difficulty providing a completed building’s area during the mid and late phases, which is particularly notable in the mid phase. The visualization results indicate that the automatic correction algorithm can obviously correct pixels during the mid and late phases, which is particularly notable in the mid phase. Despite the partial destruction of buildings, with walls broken and debris scattered, some characteristic features are still retained. However, conventional semantic segmentation algorithms fail to achieve complete extraction of the buildings. Moreover, since the buildings have not been destroyed, they cannot be extracted using the classification standards for bare land. Thus, a single semantic segmentation network alone proves insufficient in complex natural disaster scenarios. Automatic correction serves as an essential supplement by enhancing the delineation of isolated and fragmented building features, which is achieved through the exploration of relationships among adjacent video frames and the extraction of relevant motion information.

**Figure 18:**
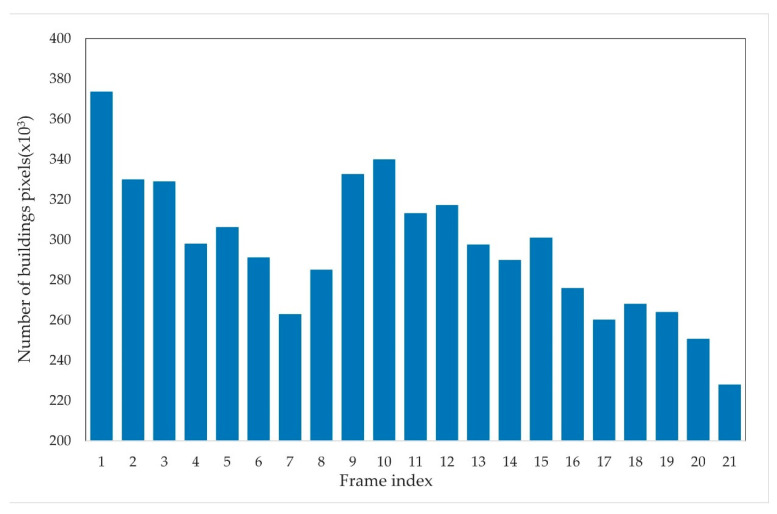
Building pixel counts in initial semantic segmentation results across 21 frames.

**Figure 19:**
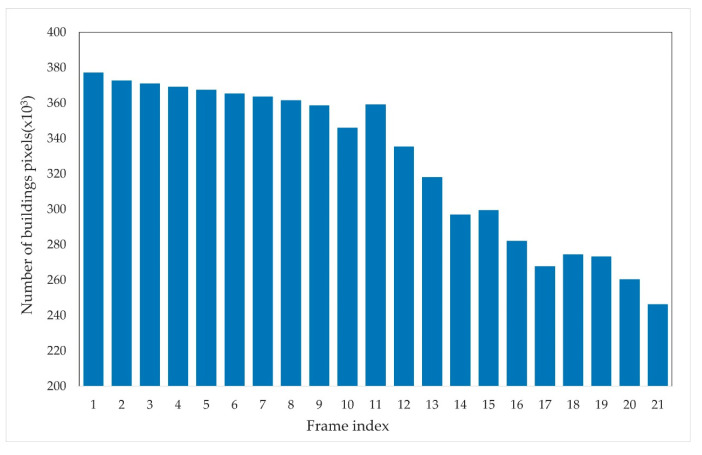
Building pixel counts in corrected semantic segmentation results across 21 frames.

Following the visual assessment, the following section presents a detailed accuracy comparison. The bar charts in Figure 18 and Figure 19 illustrate the changes in the number of building pixels derived from the initial semantic segmentation results and corrected results across different frames.

In Figure 18, the number of building pixels between frames shows no obvious regularity and exhibits irregular fluctuations. The number of building pixels in the first frame reaches a peak of about 373,736 but decreases gradually in the subsequent frames, dropping to 263,053 by the seventh frame, showing a significant downward trend with a pixel number change of 110,683. Notably, these frames are from the early to middle stages when the impact of the tsunami should be minimal, indicating that the initial semantic segmentation results may contain substantial errors. From the seventh to the twenty-first frame, the number of building pixels displays a parabolic shape, increasing up to the tenth frame and then decreasing until the end of the video, with the lowest value appearing in the twenty-first frame. These irregular fluctuations do not match the actual changes in buildings during the tsunami, indicating the semantic segmentation model’s insufficient accuracy in handling complex scenes, leading to inaccurate building recognition.

In contrast, Figure 19 shows the statistics of building areas after correction. The corrected results generally display a decreasing trend in the number of building pixels across the 21 key frames, consistent with the impact of the tsunami. In the early stages, the number of building pixels remains relatively stable with minor variations, continuing into the later stages. During the middle stages, the number of building pixels shows a significant decreasing trend, corresponding to the period when the tsunami exerts the most substantial impact, leading to severe damage to buildings. For example, the number of building pixels decreases from 346,035 in the tenth frame to 297,002 by the fourteenth frame, with a building damage rate of 14.2% in a short period.

In summary, the initial semantic segmentation results are unreliable, showing inconsistent and fluctuating building area statistics that do not accurately reflect the tsunami’s impact. The corrected results, however, display a clear improvement, aligning more closely with the actual building damage observed during the disaster, particularly during its peak impact. This demonstrates the correction method’s effectiveness in enhancing the accuracy of the segmentation, providing a more reliable representation of the changes in building areas. 

#### 3.2.5. Comparison of Different Building Extraction Algorithms

To demonstrate the advancement of the proposed model, we compared it with a state-of-the-art model across three distinct disaster periods (as shown in Table 7, Table 8 and Table 9). The comparison model selection process is as follows: Because the overall structure of our model is based on the SegNet [49] structure, the novel and improved SegFormer [59] is chosen as the comparison. UNet++ [60] and Swin-Unet [61] are classic U-shaped structures in building extraction, which have been widely used in research and industry. PFNet [62] has achieved significant results in remote sensing image segmentation in recent years.

The evaluation criteria include the intersection over union (IoU), kappa coefficient, F1 score, and overall accuracy (OA). These metrics are defined as follows:

(12) $$IoU=\frac{TP}{TP+FP+FN}$$

(13) $$Kappa=\frac{P_{0}-P_{c}}{1-P_{c}}$$

(14) $$P_{0}=\frac{TP+TN}{TP+TN+FP+FN}$$

(15) $$P_{c}=\frac{\left(TP+FP\right)\left(TP+FN\right)+(FN+TN)(FP+TN)}{{\left(TP+FP+TN+FN\right)}^{2}}$$

(16) $$F1-score=\frac{2\times Precision\times Recall}{Precision+Recall}$$

(17) $$Precision=\frac{TP}{TF+FP}$$

(18) $$Recall=\frac{TP}{TP+FN}$$

(19) $$OA=\frac{TP+TN}{TP+TN+FP+FN}$$

where true positive (TP) denotes the number of pixels correctly classified as buildings, true negative (TN) represents the number of pixels correctly classified as other objects, false positive (FP) indicates the number of pixels incorrectly classified as buildings, and false negative (FN) denotes the number of pixels incorrectly classified as other objects. 

Comparing these three tables, our method performs excellently across all three disaster periods, significantly surpassing the baseline model SegNet in all precision metrics and exceeding other comparative algorithms in key accuracy indicators. In the early stages of the disaster, our method achieves the highest scores in IoU, F1, and OA metrics, followed by the SegFormer algorithm. Specifically, our model achieves a 1.31%, 2.65%, and 0.95% improvement in the IoU, F1 score, and OA, respectively, compared to SegFormer. Although the kappa coefficient of our model is slightly lower than that of SegFormer, the difference is only 0.01. During the mid-disaster period, our algorithm continues to achieve the highest scores in IoU, F1, and OA metrics, with values of 73.50%, 83.38%, and 86.62%, respectively. In comparison, other algorithms show varying degrees of performance. PFNet ranks second in the IoU metric with a value of 71.70%, while UNet++ ranks second in the F1 and OA metrics, trailing our algorithm by 0.76% and 1.45%, respectively. SegFormer has the highest kappa coefficient at 0.76, which is 0.04 higher than our algorithm. This result highlights the instability of the four comparative algorithms. The comparison results of different accuracy indicators in the late disaster period are consistent with those in the mid-disaster period. Our algorithm still achieves the highest scores in IoU, F1, and OA metrics, while the kappa coefficient is only 0.01 lower than the highest values of Unet++ and SegFormer.

Overall, our method demonstrates outstanding performance across all three stages, accurately capturing diverse building information with higher IoUs, F1 scores, and OAs. Moreover, as the image complexity increases, our model maintains high performance, indicating that the SegNet with added correction modules exhibits greater robustness and accuracy in complex dynamic scenes. 

Since rapid building extraction in disaster scenarios is crucial, we also measured the computation time of the aforementioned algorithms, as shown in Table 10. The comparative experiments were conducted in the computational environment detailed in Table 2. The baseline model SegNet has the shortest runtime. Our improved algorithm has a slightly longer computation time, increasing by 0.27 h compared to the baseline model. PFNet has a relatively short runtime but still takes one hour more than our algorithm. UNet++ exhibits the longest runtime at 5.63 h, which is more than twice that of our algorithm. As we know, the primary strategies for improving the accuracy of deep learning models involve either increasing the training samples or the model complexity. However, these optimization strategies are often limited in disaster scenarios, which underscores the advantages of our algorithm.

## 4. Discussion

Extracting buildings accurately from remote sensing images, particularly in natural hazard scenarios, has always presented considerable challenges due to the diverse sizes and shapes of buildings and the complication of similar-looking objects like bare land. The method proposed in this paper leverages data specific to the current research context. It facilitates the rapid construction of small sample datasets and adaptively identifies erroneous pixels by monitoring for changes in building areas. Subsequently, the method utilizes optical flow information from adjacent video key frames to estimate the positions of corresponding pixels in the corrected image. This approach ensures the quick extraction of semantic information and enables the automatic correction of erroneous pixel semantics. Although some recent studies have reported combined approaches of a prediction module with a deep residual refinement module [63,64,65], significant differences can be found between these approaches and ours.

(1) This proposed algorithm does not require third-party building training datasets or significant memory resources to train large models. Therefore, it is simple to operate, highly applicable, and easy to promote and implement, offering a novel approach for the intelligent extraction of information from disaster video data.
(2) The proposed algorithm has a significant time advantage. For semantic segmentation, the annotation process is extremely costly, as each pixel must be labeled. Statistics indicate that annotating a single cityscape image size of 2048×1024 takes 1.5 h, and for images captured under adverse weather conditions, it can take up to 3.3 h. In contrast, the total calculation time for all video data using the proposed algorithm is only 6.6 h, clearly demonstrating its time efficiency.

Although the proposed method achieves impressive results in building extraction, some limitations exist in the model.

**Figure 20:**
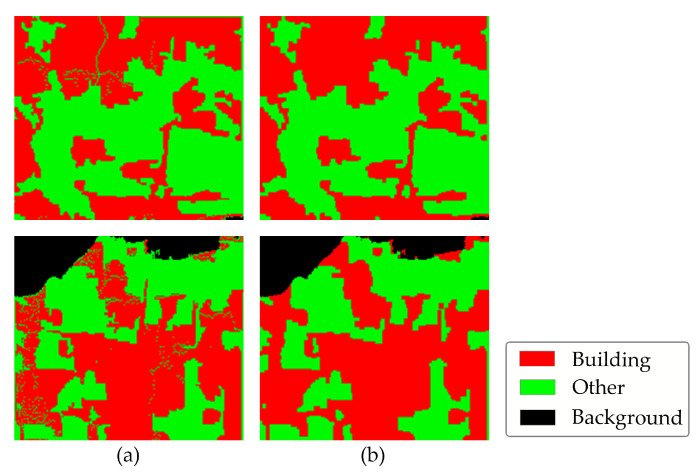
Examples of cracked images and the corresponding reconstructed images: (**a**) cracked images; (**b**) reconstructed images.

(1) As can be seen in Figure 20, in some particular areas, “many cracks” appeared in the building correction results. The issue arises from the nature of optical flow values. The movements of pixels in both the horizontal and vertical directions, as estimated by the optical flow method, do not always result in integer values. Consequently, the positions of the corresponding pixels in the corrected image—calculated as the sum of the optical flow values and the corresponding row and column numbers of the pixels—are not necessarily integers. However, these positional values must be rounded to the nearest integers, as they represent row and column numbers of pixels. This rounding can result in some rows and columns lacking semantic segmentation information due to the approximation of precise locations. In this research, we assigned the values of the nearest pixels to these missing pixels to achieve the final semantic segmentation information.

(2) From the interpretation results, this study extracts objects with any building attributes as buildings, including intact, semi-destroyed, and destroyed buildings. Assessing the extent of building damage is a critical metric in disaster evaluation; however, this study does not distinguish between disaster-damaged and non-damaged buildings. As a continuation of this work, additional data sources, e.g., the damage levels of houses, as well as the fusion of hand-crafted features and CNN-learned features, could be considered for improving this limitation. To address this limitation, future work could incorporate additional data sources, such as the damage levels of buildings, and utilize a fusion of hand-crafted features and CNN-learned features.
(3) The motion information of pixels in the proposed automatic correction module is primarily calculated using the optical flow algorithm with total variation and sparsity (OFATS). This algorithm is a dense optical flow method, which calculates the displacement of each pixel to form a dense optical flow field. Within this dense optical flow field, pixel-level motion information can be extracted from any region of the image, providing a high-precision foundation for our correction algorithm. However, since the algorithm needs to find corresponding points for each pixel across two or more images to calculate their displacement, it requires significant computational resources and is also limited by the motion amplitude between corresponding pixels in the images. If the motion between corresponding pixels is too large, the OFATS algorithm may fail to accurately estimate the optical flow for certain pixels, affecting the accuracy of the entire optical flow field and, ultimately, the precision of the correction module. This limitation restricts the usability of the proposed algorithm. In future research, we could consider further developing the automatic correction module based on a sparse optical flow estimation algorithm. By improving it to use the motion information from a limited number of corresponding points between two or more images, it could accurately estimate the motion information of pixels within the region of interest. This way, the automatic correction module would be able to provide robust motion information, even in scenarios with significant motion changes.

## 5. Conclusions

In this paper, we proposed a new semi-automatic building extraction method for natural disaster scenes using satellite video data. This method addresses the challenges of heavy dependence on training data and inaccurate building extraction, especially for buildings with complex shapes. This proposed method comprises two modules: the prediction module and the automatic correction module. The prediction module utilizes a limited training dataset generated in real-time to pre-train the classical semantic segmentation algorithm, SegNet, to obtain the initial building extraction results. Subsequently, the automatic correction module refines the semantic information of erroneous pixels based on the optical flow estimation results, enhancing the accuracy of building extraction. The experimental results obtained using satellite video data from the Indonesia tsunami demonstrate that the improved method outperforms the base network in terms of accuracy. Furthermore, the proposed method significantly surpasses four state-of-the-art methods in both computing time and building extraction accuracy.

## Figures and Tables

**Table 1:** Abbreviations and corresponding definitions in the automatic correction process.

Abbreviation	Definition	Abbreviation	Definition	Abbreviation	Definition
P	The set of erroneous pixels in the semantic segmentation result	$S_{e}$	Semantic segmentation information with erroneous pixels	$F_{e}$	Key frame corresponding to *S_e_*
		$S_{c}$	Accurate semantic segmentation information for correcting	$F_{c}$	Key frame corresponding to *S_c_* for correcting

**Table 2:** Configuration of the experimental environment.

Parameters	Value
CPU	Intel Xeon (R) E5-240
GPU	TITAN V
Memory	128 G
Operating System	Ubuntu 20.04 LTS
Framework	Pytorch 2.3.0
Python version	3.8.5

**Table 3:** Confusion matrices, along with indexes of representative data.

Frame		Ground Truth			Kappa	OA (%)
		Building	Other	UA (%)		
219	Building	9264	166	98.24	0.95	97.54
	Other	237	6780	96.62		
	PA (%)	97.51	97.61			
319	Building	12,396	36	99.71	0.98	99.26
	Other	105	6539	98.42		
	PA (%)	99.16	99.45			
409	Building	5690	4	99.93	0.93	96.60
	Other	580	10,662	94.84		
	PA (%)	90.75	99.96			

**Table 4:** Confusion matrix of initial extraction results for three frames.

Frame		Ground Truth			Kappa	OA (%)
		Building	Other	UA (%)		
179	Building	292,049	37,924	88.51	0.73	87.48
	Other	76,923	510,354	86.90		
	PA (%)	79.15	93.08			
339	Building	281,948	58,052	82.93	0.65	83.67
	Other	91,753	485,495	84.11		
	PA (%)	75.45	89.32			
439	Building	185,837	64,951	74.10	0.63	85.67
	Other	66,554	600,034	90.02		
	PA (%)	73.63	90.23			

**Table 5:** Correction process for highlighted area in rose from Figure 13.

	Operations	Frame 179	Frame 339	Frame 439
**Identifying erroneous information**	Assessment of the overall change trend	$S_{f_{1}}=18,294$ $S_{f_{21}}=19,335$	$S_{f_{1}}=15,631$ $S_{f_{21}}=19,212$	$S_{f_{1}}=5277$ $S_{f_{21}}=1726$
		$Diff=S_{f_{1}}-S_{f_{21}}<0$	$Diff=S_{f_{1}}-S_{f_{21}}<0$	$Diff=S_{f_{1}}-S_{f_{21}}>0$
	Calculation of the building area in the selected patch	$S_{f_{1}}=18,294$ $S_{f_{2}}=15,615$ $S_{f_{3}}=16,301$	$S_{f_{9}}=15,882$ $S_{f_{10}}=18,510$ $S_{f_{11}}=15,795$ $S_{f_{12}}=16,647$	$S_{19}=1856$ $S_{f_{20}}=3396$ $S_{f_{21}}=1726$
	Determination of images with erroneous pixels	$S_{f1}-S_{f2}>500$ and $S_{f3}-S_{f2}>500$	$S_{f10}-S_{f9}>500$ and $S_{f10}-S_{f11}>500$	$S_{f20}-S_{f19}>500$ and $S_{f20}-S_{f21}>500$
		$S_{f1}-S_{f3}>500$	$S_{f10}-S_{f12}>500$	$S_{f21}-S_{f19}<500$
		*S_e_ =*$S_{f1}$ *F_e_ = F*_1_ *S_c_* = $S_{f2}$ *F_c_ = F*_2_	*S_e_* = $S_{f10}$ *F_e_ = F*_10_ *S_c_ =* $S_{f9}$ *F_c_ = F*_9_	*S_e_ =*$S_{f20}$ *F_e_ = F*_19_ *S_c_ =* $S_{f19}$ *F_c_ = F*_18_
**Correcting errors**	Extraction of erroneous pixels’ coordinates	$N_{1}=(x_{i},y_{j})$	$N_{2}=(x_{i},y_{j})$	$N_{3}=(x_{i},y_{j})$
	Optical flow information	$I_{1}\left(f_{2}\to f_{1}\right)=\left(u_{i},v_{j}\right)$	$I_{2}\left(f_{9}\to f_{10}\right)=\left(u_{i},v_{j}\right)$	$I_{3}\left(f_{19}\to f_{20}\right)=\left(u_{i},v_{j}\right)$
	Corresponding coordinates in corrected images	$N_{1}^{\prime }=N_{1}+I_{1}$	$N_{2}^{\prime }=N_{2}+I_{2}$	$N_{3}^{\prime }=N_{3}+I_{3}$
	Replacement of incorrect semantic segmentation	$S_{c}\;of\;N_{1}^{\prime }\to S_{e}$	$S_{c}\;of\;N_{2}^{\prime }\to S_{e}$	$S_{c}\;of\;N_{3}^{\prime }\to S_{e}$

**Table 6:** Confusion matrices for automatic correction results of the three frames.

Frame		Ground Truth			Kappa	OA (%)
		Building	Other	UA (%)		
179	Building	314,186	43,585	87.82	0.77	89.28
	Other	54,786	504,693	90.21		
	PA (%)	85.15	92.05			
339	Building	307,946	65,755	82.40	0.72	86.62
	Other	56,982	486,565	89.52		
	PA (%)	84.39	88.09			
439	Building	207,201	45,190	82.09	0.70	87.99
	Other	65,010	599,975	90.22		
	PA (%)	76.12	92.99			

**Table 7:** Comparison of various building extraction algorithms based on an early phase frame.

Model	IoU (%)	Kappa	F1 (%)	OA (%)
Swin-Unet (2021) [61]	73.25	0.74	81.25	86.27
UNet++ (2018) [60]	72.65	0.73	80.90	86.15
PFNet (2021) [62]	73.15	0.72	80.65	85.60
SegFormer (2021) [59]	73.96	0.74	81.59	86.70
SegNet (2016) [49]	69.30	0.68	80.05	84.52
Ours	75.27	0.73	84.24	87.65

**Table 8:** Comparison of various building extraction algorithms based on a middle-phase frame.

Model	IoU (%)	Kappa	F1 (%)	OA (%)
Swin-Unet (2021) [61]	71.14	0.73	81.51	84.03
UNet++ (2018) [60]	71.39	0.72	82.62	85.17
PFNet (2021) [62]	71.70	0.72	81.84	84.73
SegFormer (2021) [59]	70.73	0.76	80.20	83.82
SegNet (2016) [49]	65.30	0.65	79.01	83.67
Ours	73.50	0.72	83.38	86.62

**Table 9:** Comparison of various building extraction algorithms based on a late-phase frame.

Model	IoU (%)	Kappa	F1 (%)	OA (%)
Swin-Unet (2021) [61]	70.34	0.70	75.52	86.07
UNet++ (2018) [60]	70.95	0.71	76.77	87.28
PFNet (2021) [62]	70.67	0.67	77.12	86.46
SegFormer (2021) [59]	70.19	0.71	75.84	85.94
SegNet (2016) [49]	60.36	0.63	73.86	85.67
Ours	72.15	0.70	78.99	87.99

**Table 10:** The comparison of running times for different building extraction networks.

	Swin-Unet	UNet++	PFNet	SegFormer	SegNet	Ours
Time (h)	4.97	5.63	3.42	4.76	2.47	2.74

## Data Availability

The original contributions presented in the study are included in the article, further inquiries can be directed to the corresponding author.
